# Collagen-Embedded Three-Dimensional Spheroid Model Based on Breast and Cervical Cancer for Drug Screening Application

**DOI:** 10.7759/cureus.93920

**Published:** 2025-10-06

**Authors:** Pallavi Kulkarni, Surbhi Khare, Swagata Bramhachari, Saikat Das, Neelkamal Kapoor, Sudhir K Goel, Prasoon Kumar, Ashok Kumar, Neha Arya

**Affiliations:** 1 Department of Biochemistry, All India Institute of Medical Sciences, Bhopal, Bhopal, IND; 2 Department of General Surgery, All India Institute of Medical Sciences, Bhopal, Bhopal, IND; 3 Department of Radiotherapy, All India Institute of Medical Sciences, Bhopal, Bhopal, IND; 4 Department of Pathology and Laboratory Medicine, All India Institute of Medical Sciences, Bhopal, Bhopal, IND; 5 Department of Biochemistry, T.S. Misra Medical College and Hospital, Lucknow, IND; 6 Department of Biotechnology and Medical Engineering, National Institute of Technology, Rourkela, Rourkela, IND; 7 Department of Translational Medicine, All India Institute of Medical Sciences, Bhopal, Bhopal, IND

**Keywords:** 3d spheroids, breast cancer, cervical cancer, collagen, drug testing platform, in vitro tumor model

## Abstract

Background

Despite advancements in treatment protocols, breast and cervical cancer are the leading causes of cancer-associated deaths in Indian women. Therefore, there is a need to develop more effective treatment strategies and better tumor models to test novel therapeutics. This study reports the development of a bioengineered three-dimensional (3D) model that can mimic in vivo tumors and be used to understand disease pathophysiology, as well as for drug testing/screening applications.

Methodology

The liquid overlay method was used to generate 3D spheroids of uniform size, based on breast (MDA-MB-231) and cervical (HeLa and CaSki) cancer cell lines. After this, they were embedded in collagen type I. The collagen-embedded spheroids were then subjected to live/dead staining, viability study, and expression of epithelial-mesenchymal transition (EMT) markers at predetermined time points. The 3D spheroids were also subjected to anti-cancer drug testing, followed by a viability assay. The software used for analysis was GraphPad Prism 8.4 (Dotmatics, Boston, Massachusetts, United States) and MS Excel (Microsoft Corporation, Redmond, Washington, United States).

Results

Uniform-sized, single spheroids for three different cell lines, MDA-MB-231, HeLa, and CaSki, were successfully generated using the liquid overlay technique. There was a significant increase in the viability of 3D spheroids after day 3 of incubation when compared with day 1. Live/dead staining showed the presence of dead cells in the core of the 3D spheroids. Further, EMT markers such as twist, N-cadherin, and fibronectin were found to be elevated in the case of 3D spheroids as compared to the two-dimensional (2D) culture of cancer cells. Additionally, the IC50 values of MDA-MB-231, HeLa, and CaSki spheroids following treatment with an anti-cancer drug, cisplatin, were found to be approximately four to five-fold higher than in the 2D culture.

Conclusion

The collagen-embedded 3D spheroid model is a robust model that can be used for the generation of 3D spheroids based on various cancer cell lines, recapitulating the properties of solid tumors in vivo. The model further demonstrates its potential to be used as a drug screening platform and can be used for the generation of patient tumor-derived 3D spheroids in the future.

## Introduction

Breast cancer and cervical cancer are major causes of cancer-related deaths in women [1]. While current treatment methods have shown improvement, recurring disease is still a big challenge. The cause of the recurrence is mainly due to metastasis, resistance to therapy, tumor heterogeneity, and the tumor microenvironment (TME), which promotes the cancer cells' survival and proliferation [2]. Therefore, there is a need to develop novel treatment strategies for the management of breast and cervical cancer. Towards this, drug development and screening are typically carried out on two-dimensional (2D) tissue culture polystyrene (TCPS) and xenograft mouse models. However, due to the disadvantages associated with conventional drug screening models, there has been a paradigm shift towards the development of better in vitro three-dimensional models that can mimic the in vivo tumors and be used to understand disease pathophysiology as well as for drug testing/screening studies.

For the generation of a three-dimensional (3D) spheroid model, replicating the features of the TME is necessary. The TME plays a vital role in tumor progression, drug response, and metastasis [3]. It includes various components, such as stromal cells, immune cells, stem cells, extracellular matrix (ECM), and signalling molecules [4]. These factors together affect tumor progression by changing the stromal topography, aiding the invasion of cancer cells, and limiting drug penetration, allowing chemoresistance to tumors over time [5,6].

Collagen, a major component of the ECM, is crucial for defining the tumor structure within the TME. Matrices that are rich in collagen provide structural support, affect cell adhesion, and shape important signalling pathways related to tumor growth and metastasis [7,8]. The collagen matrix acts as a barrier that impacts the drug availability for cancer cells, making these models valuable for assessing the therapeutic effectiveness in environments similar to in vivo tumors [9]. Incorporating the intricacies of the TME and ECM into 3D culture systems can help better understand cancer biology, uncover potential therapeutic targets, and refine drug screening methods [10].

Within various 3D culture systems, 3D spheroids are widely utilized 3D cell culture models due to their cell aggregate formation and development into multicellular structures. This spherical arrangement imparts unique properties to cancer cells, such as transport gradients and stratified organization [11]. Various 3D culture techniques generate spheroids, including the liquid overlay method and scaffold-based methods [12]. The most widely and documented use method is the liquid overlay technique, which uses treated culture vessels to prevent cells from adhering to their surface, in turn promoting the spherogenicity property of cancer cells to form spheroids [13,14].

Our study mainly focuses on developing and characterizing a collagen-embedded uniform-sized 3D spheroid-based tumor model from breast and cervical cancer cell lines. The developed spheroids were characterized for various properties of solid tumor and further assessed for drug response at pre-defined time points. The findings highlight the relevance of 3D tumor models in studying the breast and cervical cancer progression and drug responsiveness, bridging the gap between conventional in vitro studies and in vivo tumor behaviour. The study also emphasises the potential of the developed model as a drug screening platform.

The study's data have been presented in the form of a poster at the 41st Annual Conference of the Indian Association for Cancer Research (IACR), held in Noida, India, in March 2021.

## Materials and methods

Cell culture on 2D TCPS

To develop 3D bioengineered tumor models for breast and cervical cancer, the cell lines MDA-MB-231 and CaSki were purchased from the American Type Culture Collection (ATCC), Manassas, Virginia, United States, and the cell line HeLa was purchased from the European Collection of Authenticated Cell Cultures (ECACC), Salisbury, Wiltshire, United Kingdom. The cells were maintained at 5% CO2 and 37°C in minimum essential media (MEM) (Thermo Fisher Scientific Inc., Waltham, Massachusetts, United States) with 10% fetal bovine serum (FBS) (Thermo Fisher Scientific Inc.) and 1% penicillin-streptomycin (Pen-Strep) (Thermo Fisher Scientific Inc.). Cells were trypsinized using 0.25% trypsin-ethylenediamine tetraacetic acid (EDTA) solution, and before cell seeding, the cell number was counted using a Neubauer chamber.

Isolation and characterization of collagen type I from the Achilles tendon

Collagen type I extraction was carried out using the Achilles tendon of a one-year-old male goat obtained from a local slaughterhouse using a previous protocol [15]. The tendon was first washed with autoclaved water to remove any residual blood and impurities before being finely chopped with sterile blades. It was then incubated in 0.5 N acetic acid at 4°C for six hours, followed by overnight digestion with 2% pepsin. The processed mixture was subsequently filtered, and collagen precipitation was achieved using 30% sodium chloride (NaCl). The precipitated collagen was dissolved in a minimal volume of 0.5 N acetic acid and dialysed (12-15 KDa) against 10 mM HCl at 4°C for three days, after which it was stored under the same conditions. Isolated collagen was characterised using Sodium dodecyl sulfate polyacrylamide gel electrophoresis (SDS-PAGE). For this, the commercially available collagen type I standard and the extracted collagen were loaded on a 4% stacking and 8% resolving polyacrylamide gel. After the electrophoresis, the gel was stained with Coomassie Brilliant Blue stain, followed by destaining. The comparative bands of isolated and standard collagen were observed on the digital image of the gel. To assess the concentration of isolated collagen type I, the extracted sample was incubated with 50 µM Sirius red dye for 30 minutes. The resulting pellet was dissolved in 0.1 N potassium hydroxide, and absorbance was measured at 540 nm using a microplate reader (EON; BioTek Instruments, Winooski, Vermont, United States). This absorbance was used to calculate the concentration of collagen type I using the standard curve generated with the commercial collagen type I (Merck KGaA, Darmstadt, Germany).

Generation of collagen-embedded bioengineered 3D spheroids using the liquid overlay method

The liquid overlay technique relies on a non-adhesive surface to prevent cell attachment to the substrate, thereby enhancing cell-cell interactions. This approach promotes cell aggregation, resulting in spheroid formation. A non-adhesive surface was created by applying a thin layer of 1% w/v agarose (Merck KGaA) to the wells of 96-well tissue culture plates. Using this method, spheroids of varying sizes were generated in 96-well plates by seeding cells at different densities (5x10^3^, 10x10^3^, 25x10^3^, and 50x10^3^ cells per well). After 24 hours of incubation, the cell aggregates were embedded in a type I collagen pre-gelation mixture (collagen type I, 0.1N NaOH, 10X phosphate-buffered saline (PBS), and water). The cell aggregates within the pre-gelation mixture were incubated at 37°C for 45 minutes to induce hydrogel formation. Following hydrogel formation, growth media were layered over the hydrogels. These embedded spheroids were maintained for six days, with fresh media added every alternate day till day 6 [15].

Live/dead staining 

The 3D spheroids were assessed for their necrotic core using live/dead staining. The staining solution consisted of 8 µl of fluorescein diacetate (FDA, 5 mg/ml) and 50 µl of propidium iodide (PI, 2 mg/ml) in 5 ml of serum-free media. At the end of six days of incubation, the collagen-embedded spheroids were incubated with the staining solution at room temperature for 10 minutes. Following incubation, the staining solution was removed, and the embedded spheroids were washed twice with PBS and imaged using a Nikon Eclipse Ti-S inverted microscope, 488/545 nm and 594/660 nm (Nikon Corporation, Shinagawa, Tokyo, Japan).

WST-1 viability assay

For the viability test, the WST-1 assay was performed at the end of day 1, day 3, and day 6 of incubation. WST-1 assay relies on the cleavage of stable tetrazolium salt WST-1 to form a soluble formazan crystal at the cell surface. At the end of days 1, 3, and 6, the collagen-embedded spheroids were incubated with the WST-1 reagent (Merck KGaA) in a 1:10 dilution for two hours. Thereafter, 100 µL of the medium/WST-1 solution was transferred to a 96-well microtiter plate to measure optical density at 450 nm (OD450), with the 650 nm reference wavelength, using a microplate reader (BioTek Instruments).

Expression of epithelial-mesenchymal transition (EMT) markers using quantitative reverse transcription-polymerase chain reaction (qRT-PCR)

For the determination of expression of EMT markers, RNA was isolated from collagen-embedded spheroids using a TRIzol reagent (Thermo Fisher Scientific Inc.), followed by DNAse treatment and reverse transcription using an iScript cDNA Synthesis System (Bio-Rad Laboratories, Inc., Hercules, California, United States) according to the manufacturer's instructions. Each biological replicate was considered for quantitative PCR reactions for both target genes and the housekeeping gene. The SYBR Green PCR master mix (Bio-Rad Laboratories, Inc.) and the primers (Table 1) were used to set up the reactions according to the manufacturer's instructions. The mRNA expression levels of various EMT markers were normalised with the expression of the housekeeping gene, i.e., 18S rRNA, and were compared to the respective 2D TCPS. The thermocycler temperature profile is mentioned in Table 2.

**Table 1:** Primer/oligos sequences used in qRT-PCR qRT-PCR: quantitative reverse transcription-polymerase chain reaction

S. No.	Gene Name	Primer Sequence 5’-3’	
1.	18S rRNA	F	GGTAACCCGTTGAACCCCAT
		R	CAACGCAAGCTTATGACCCG
2.	Vimentin	F	CTGCCAACCGGAACAATGAC
		R	CCACTTCACAGGTGAGGGAC
3	TWIST1	F	CCACTGAAAGGAAAGGCATC
		R	CTATGGTTTTGCAGGCCAGT
4	Fibronectin	F	CTGAAAGACCAGCAGAGGCA
		R	GTGTAGGGGTCAAAGCACGA
5	N-Cadherin	F	GTATCCGGTCCGATCTGCAG
		R	GAGCTGTGGGGTCATTGTCA

**Table 2:** Temperature profile for qRT-PCR for expression of EMT marker qRT-PCR: quantitative reverse transcription polymerase chain reaction; EMT: epithelial–mesenchymal transition

S. No.	Steps	Temperature	Time	Cycles
1	Initial Denaturation	95°C	3 mins	1
2	Denaturation	95°C	30 sec	40
3	Annealing	55°C	30 sec	
4	Extension	72°C	1 min	
5	Melting Curve	65°C	5 sec	1
6		95°C	50 sec	1

Drug testing/screening on collagen-embedded 3D spheroids

The collagen-embedded 3D spheroids were treated with commercially available chemotherapeutic agents, doxorubicin and cisplatin (Merck KGaA), at concentrations ranging from 0 to 500 μM for 24 hours. Similarly, cells cultured on 2D TCPS were treated with the same chemotherapeutic agents, at concentrations ranging from 0 to 50 μM. Following treatment, the WST-1 assay was used to assess spheroid viability, and the absorbance was measured at 450 nm. Viability percentages were calculated relative to the untreated control, and absolute IC50 values were determined using non-linear regression analysis in GraphPad Prism software v8.4 (Dotmatics, Boston, Massachusetts, United States).

Statistical analysis

Results represent the mean values of replicates of spheroid cultures, and error bars represent standard deviations. The statistical significance of data was evaluated using a non-parametric test for the PCR study, t-test for drug studies, and two-way ANOVA for WST-1-based growth studies, using GraphPad Prism software. P values <0.05 were considered statistically significant.

## Results

Collagen type-1 was successfully isolated from the goat’s Achilles tendon

**Figure 1:**
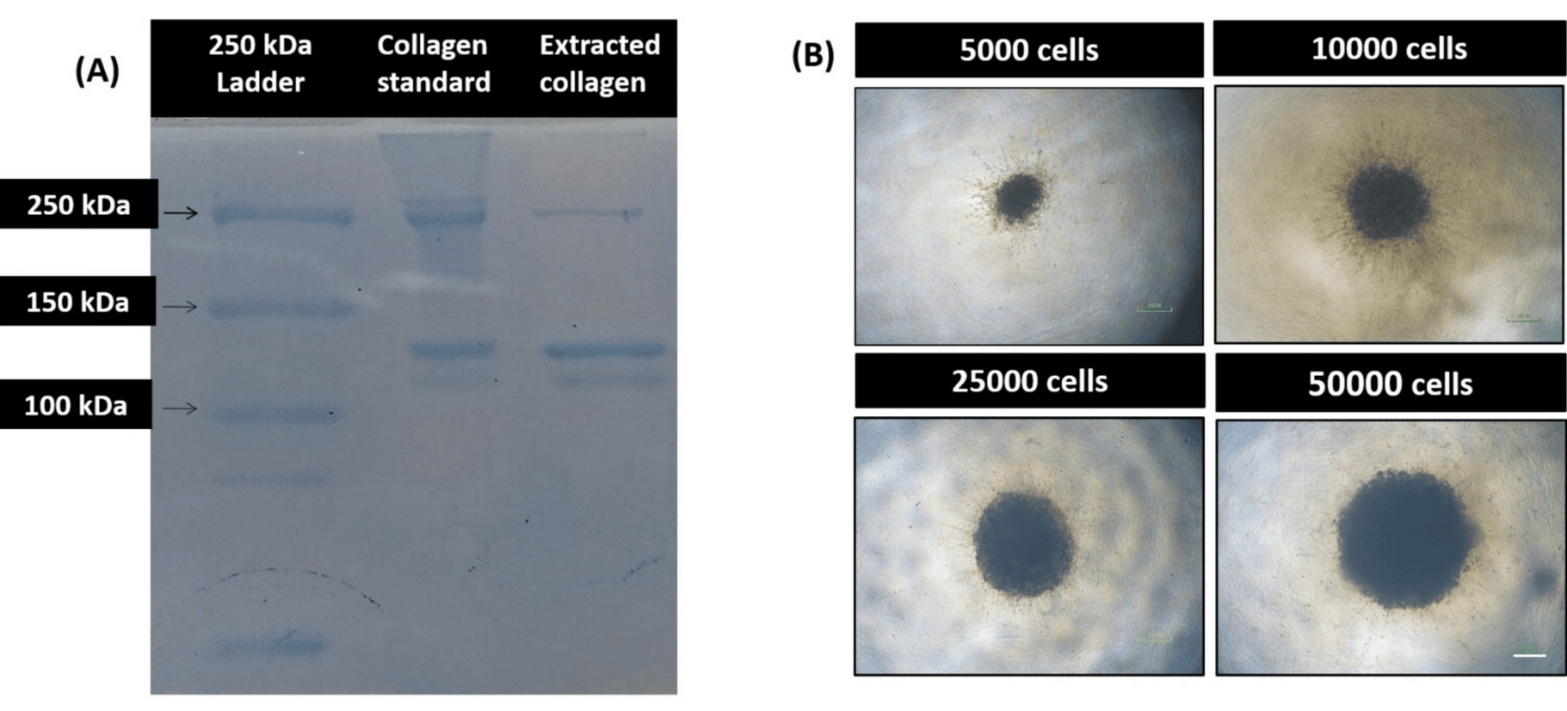
Characterization of isolated collagen and generation of collagen embedded spheroids (A) SDS-PAGE demonstrating desired bands for β, α1 & α2 chains of collagen. (B) Representative photomicrographs of collagen-embedded spheroids of 5000, 10000, 25000 and 50000 cells (Scale: 100 µm). SDS-PAGE: sodium dodecyl sulfate-polyacrylamide gel electrophoresis

To generate collagen-embedded 3D spheroids, the collagen type I was first isolated and characterised from the goat Achilles tendon. The type and purity of the isolated collagen were further checked using SDS-PAGE and the Sirius red assay (Figure 1A). The isolated collagen exhibited three clear bands at 250 kDa corresponding to the β chain, and at 120 kDa and 115 kDa corresponding to the α1 and α2 chains, respectively, similar to the commercial collagen type I, thereby confirming the isolated collagen as type I. Further, the results corroborated with a previously published study [15]. Furthermore, the concentration of the isolated collagen was calculated using the Sirius red assay with respect to the standard curve of collagen type I. The concentration was found to be between 4 and 6 mg/ml.

3D bioengineered collagen-embedded spheroids increase in size as a function of cell number and time

**Figure 2:**
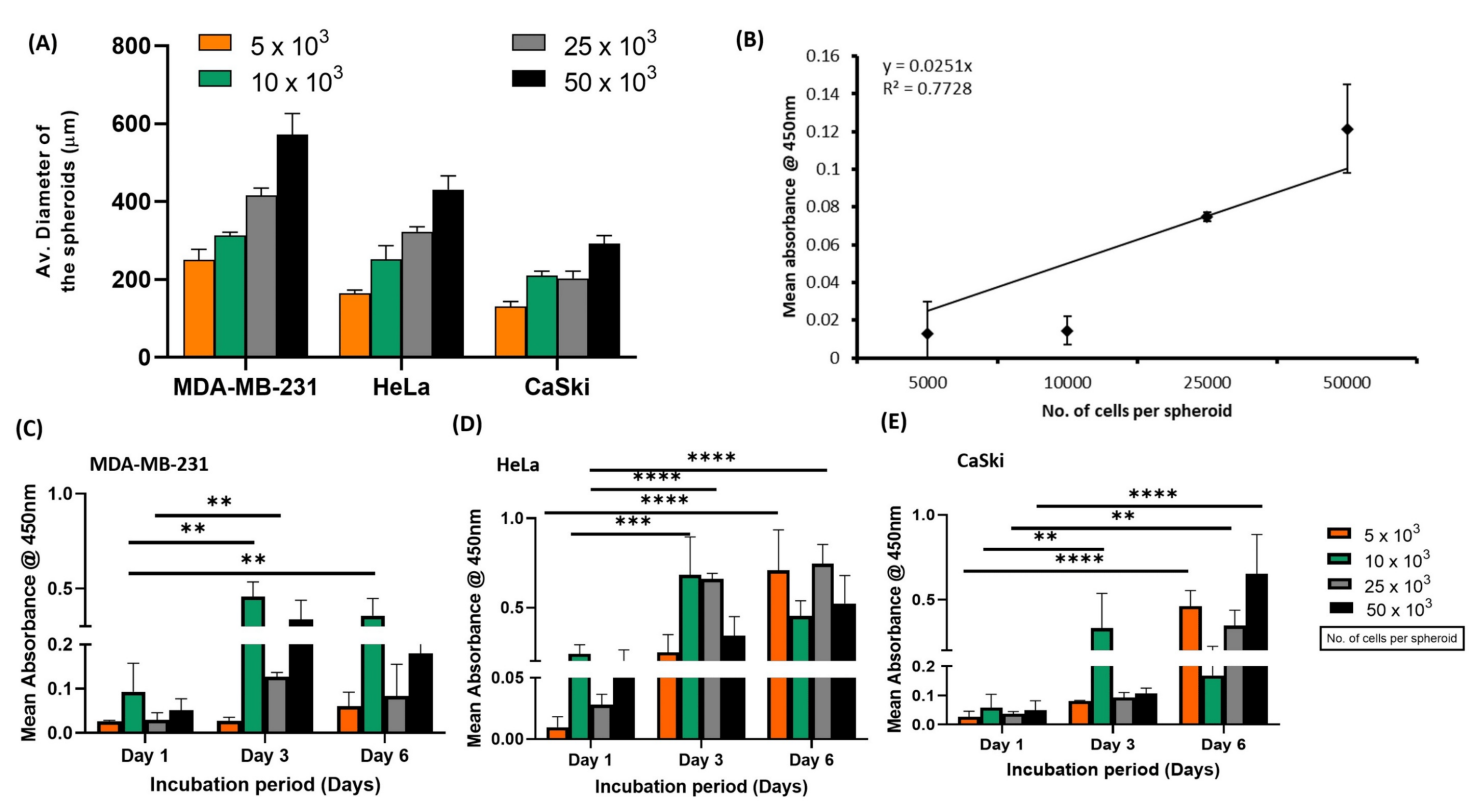
Size and viability of collagen embedded 3D spheroids based on breast and cervical cancer cells (A) Graph representing the size of the collagen-embedded 3D spheroids based on breast (MDA-MB-231) and cervical cancer (HeLa and CaSki) as a function of cell number; (B) WST-1 assay showing a linear trend, as the absorbance increases size as a function of the number of cells seeded for the generation of spheroids. Representative graphs show the growth of 3D spheroids over six days. Mean absorbance of 3D spheroids based on (C) MDA-MB-231, (D) HeLa, and (E) CaSki, as a function of cell number and time. ** P<0.001, *** P<0.0001 **** P<0.00001, level of significance when the viability at the end of day 3 and day 6 spheroids are compared with day 1.

Following the successful characterization of collagen type I, the 3D spheroids were generated using the liquid overlay method, followed by embedding in collagen (Figure 1B). The morphology and size of 3D spheroids were further characterized using light microscopy, and the average diameter of the breast cancer and cervical cancer 3D spheroids was determined at the end of six days of incubation using ImageJ software (imagej.net). The diameters of 3D spheroids were directly proportional to the cell number. At the end of day 6, the largest spheroids produced using 50000 cells had average diameters of 572±54.3µM (MDA-MB-231), 431.3±35.2µM (HeLa), and 292.1±20.99µM (CaSki). Figure 2A represents the average diameter of the generated spheroids of various cell densities at the end of day 6 of incubation. Further, the tight standard deviations suggest the uniformity in the size of the generated 3D spheroids.

Further, the 3D spheroids were assessed for their viability using the WST-1 proliferation assay. The mean absorbance at 450 nm, indicative of cell proliferation, was measured for 3D spheroids with varying cell densities (5 x 10^3^, 10 x 10^3^, 25 x 10^3^, and 50 x 10^3^ cells per spheroid) as a function of time, i.e., at the end of day 1, day 3, and day 6. At the end of day 1, absorbance levels were uniformly low across all cell densities, showing no significant variations between the groups. Further, at the end of day 2, a clear increase in cell growth (absorbance) was observed, particularly in spheroids containing 25 × 10³ and 50 × 10³ cells, indicating enhanced proliferation compared to day 1. However, at the end of day 6, absorbance showed a slight decline. As anticipated, the highest absorbance, reflecting maximum cell growth, was recorded for spheroids with 50 × 10³ cells. These findings highlight that cell proliferation in spheroids is influenced by both cell density and incubation duration, with higher cell densities promoting increased cell numbers (absorbance) over time (Figure 2B-2E).

Furthermore, to assess the scalability and consistency of spheroid formation, we examined how cell seeding density influences spheroid diameter. As illustrated in Figure 2B, a trend line was generated for cell line-based spheroids, confirming the robustness and reproducibility of this approach. The data closely followed a power-law model (R² > 0.75), exhibiting minimal variability in spheroid size. Again, the error bars account for cell counting inaccuracies inherent to hemocytometer measurements. These findings validate the ability to produce uniform samples while precisely controlling spheroid size consistently.

3D bioengineered collagen-embedded spheroids recapitulated the properties of in vivo solid tumors

**Figure 3:**
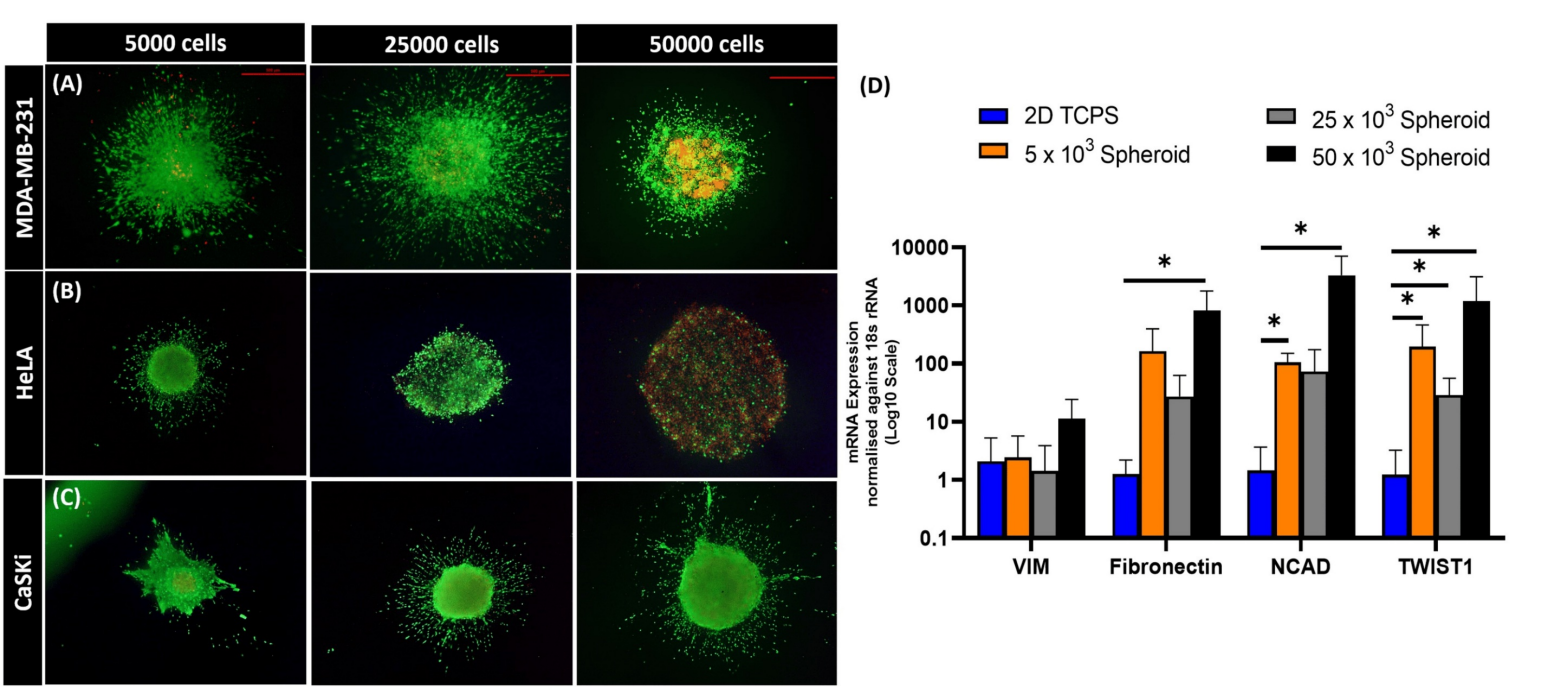
Characterization of collagen-embedded 3D spheroids Representative images of live-dead staining showing dead cells at the core of (A) MDA-MB-231, (B) HeLa, and (C) CaSki spheroids  (Scale: 500µm). (D) Representative bar graph showing mRNA expression of EMT markers in 3D spheroids (MDA-MB-231) as compared to 2D culture; gene expression was normalized with respect to 18srRNA. *P<0.05, level of significance when the mRNA expression of EMT markers in 2D culture was compared with 3D collagen-embedded spheroid culture. VIM: vimentin; FIB: fibronectin; NCAD: N-cadherin; TWIST-1: Twist-related protein 1; 3D: three-dimensional; 2D: two-dimensional; EMT: epithelial–mesenchymal transition

An important trait of solid tumors is the development of a necrotic core [16]. In this regard, the 3D spheroids were also subjected to live/dead staining with fluorescein diacetate (FDA) and propidium iodide (PI). The core of 3D spheroids consisted of dead cells and was stained red with PI, indicating the presence of a necrotic core similar to tumors in vivo (Figures 3A-3C). It was also observed that as the size of the spheroid increased, the size of the necrotic core increased. Another significant feature of the solid tumors is the overexpression of EMT and mesenchymal markers [17]. It was observed that EMT markers like twist-1, vimentin, fibronectin, and N-Cadherin were overexpressed in the collagen-embedded 3D spheroids when compared with the 2D cell culture (Figure 3D). Taken together, both the live/dead staining and the elevated EMT markers suggest tumor-like properties in the bioengineered 3D collagen-embedded spheroids.

3D bioengineered collagen-embedded spheroids show decreased sensitivity to chemotherapeutic agents compared to 2D cell culture

**Figure 4:**
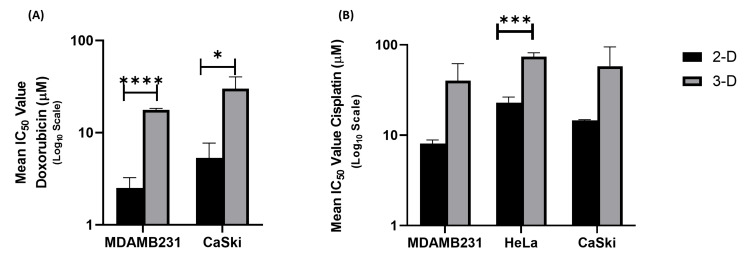
Drug response of collagen embedded 3D spheroids-based on breast and cervical cancer IC50 of collagen embedded 3D spheroids based on breast (MDA-MB-231) and cervical cancer cells (HeLa and CaSki) as compared to 2D cell culture following treatment with (A) Doxorubicin (IC50​​​​​​​ values of 3D spheroids are 17.33 µM and 32.22 µM for MDA-MB-231 and CaSki, respectively) and (B) Cisplatin (IC50​​​​​​​ values of 3D spheroids are 18.59 µM, 65.46 µM, and 15.54 µM for MDA-MB-231, HeLa, and CaSki, respectively. *P<0.05, ***P<0.0001, ****P<0.00001, level of significance in difference in IC50​​​​​​​ of 2D and 3D cultures. 2D: two-dimensional; 3D: three-dimensional

After the successful characterisation of the 3D collagen-embedded spheroids, the model was further characterised for its translational potential regarding drug screening. To address this, the collagen-embedded 3D spheroids based on MDA-MB-231, HeLa, and CaSki were treated with a range of concentrations of standard chemotherapeutic agents, doxorubicin and cisplatin. It was found that there was a significant increase in the IC50 value for the 3D spheroids compared to 2D cell culture. The IC50 values of MDA-MB-231, HeLa, and CaSki spheroids following treatment with the anti-cancer drug cisplatin were found to be 89.24 µM, 186.95 µM, and 106.67 µM, respectively, which were approximately four to five-fold higher than those of the 2D cultures. In the case of doxorubicin, the IC50 values were 17.33 µM for MDA-MB-231 and 32.22 µM for CaSki, which is approximately five to six times more than those in the 2D cultures. This particular finding validates the crucial aspect of solid tumors regarding the reduced bioavailability of chemotherapeutic molecules. Further, it also suggests that the 3D tumor architecture, along with extracellular matrix deposition, contributes to drug resistance by limiting drug accessibility and altering cellular sensitivity to chemotherapeutics (Figure 4).

## Discussion

Solid tumors such as breast and cervical cancer pose significant challenges in treatment due to the reduced bioavailability of chemotherapeutic molecules, which can limit drug penetration and effectiveness [18]. Consequently, there is an urgent need to develop a model that accurately replicates in vivo tumor conditions, enabling a deeper understanding of disease mechanisms and drug responses. 3D tumor spheroid models have initiated a paradigm shift in preclinical cancer research, as they faithfully recapitulate key aspects of the complex in vivo TME compared to cells grown on two-dimensional TCPS [19]. More specifically, they effectively mimic tumor heterogeneity and pathophysiology, making them widely accepted surrogates for drug discovery and preclinical studies, thereby bridging the gap between 2D cell cultures and in vivo models [19].

Within the TME, stromal populations and extracellular matrix contribute to EMT, stemness, and chemoresistance [20]; therefore, it is crucial to develop more realistic 3D models that have similar culturing practices and mimic the properties of in vivo tumors. Thus, this study aims to create a 3D model utilising breast cancer and cervical cancer spheroids with collagen as extracellular matrix components to understand drug sensitivity in solid tumors. In this study, the generation of 3D spheroids based on breast cancer (using MDA-MB-231 cells) and cervical cancer (using HeLa and CaSki cells) was performed using the liquid overlay technique, followed by collagen embedding of the spheroids. 3D spheroid formation using the liquid overlay method relies on the inherent capacity of cells to aggregate. Adjusting the culture medium or incorporating a matrix can enhance this process. For instance, in this study, breast and cervical cancer cells were cultured in 3D under liquid overlay conditions using the same medium as in 2D, but the addition of collagen matrix enhanced the formation of more complex structures. However, previous studies successfully generated well-defined breast and cervical cancer spheroids with distinct borders by incorporating basement membrane or Matrigel™. Additionally, various other 3D culture techniques with different matrices have been developed for spheroid generation and other applications [21,22]. The morphology of the generated 3D spheroid was evaluated using key shape parameters, such as average diameter to represent size, roundness to define shape, and spheroid count per well. It was observed that using the liquid overlay method, uniform-sized spheroids were generated, whose size was regulated as a function of the cell number. 

The bioengineered spheroid model was further evaluated for key solid tumor characteristics to confirm that the generated spheroids accurately mimic in vivo tumor conditions. The 3-D spheroids showed enhanced expression of various metastatic markers like TWIST-1, vimentin, fibronectin, and N-cadherin. Similar findings were observed in other gynaecological cancer studies with collagen matrices embedded 3D cultures [23,24]. Further, when the 3D spheroids were subjected to the live-dead staining, the collagen-embedded spheroids showed the presence of propidium iodide-stained dead core. Similarly, the glioblastoma (GBM) spheroids generated by Bach et al. showed PI-stained necrotic core when generated via low attachment TCPS plates. However, these GBM spheroids were not embedded in any matrices [25]. In yet another study, the live cells and dead core or MCF7-based collagen-embedded spheroids were shown using calcein (green) and ethidium bromide (red) stains. Here, calcein stained the metabolically active cells while the ethidium bromide stained the cells with disrupted cell membranes [26]. This validates that the generated collagen-embedded 3D tumor model replicates the natural ECM, adaptations to hypoxia due to the necrotic core, and has enhanced EMT markers, which further promote metastasis.

The main aim of the study was to establish a straightforward biomimetic 3D culture method that has an ECM component for breast and cervical cancer cell lines to use in investigating cancer biology and treatment response. The generated 3D spheroids were deemed suitable for a drug screening platform based on three key criteria i.e., spheroids must form a hypoxic core, maintain a spherical and uniform shape as a function of cell number to ensure uniform molecular diffusion, and each well must contain a single spheroid. As a proof of concept, the efficacy of doxorubicin and cisplatin was assessed in MDA-MB-231, HeLa, and CaSki spheroids generated using the liquid overlay method followed by collagen type-1 embedding, comparing outcomes with conventional 2-D culture. Previously, it has been shown that the drug response in 3D differs from 2D due to the spatial organization of spheroids [27], which restricts assay options. More suitable approaches include analyzing spheroid supernatant, such as soluble formazone (WST-1) for quantifying viability, or sectioning methods to visualize internal structures were adapted for the same. Hence, these 3D spheroids were subjected to treatment with varying concentrations of cisplatin and doxorubicin to assess the differences in drug response as compared to 2D cultures using the WST-1 assay. The results demonstrated a significant increase in the IC50 values for cisplatin and doxorubicin in the 3D spheroid models compared to their 2D counterparts. The IC50 values of MDA-MB-231, HeLa, and CaSki spheroids following treatment with cisplatin were found to be approximately four to five-fold higher than those of the 2D cultures. Similarly, in the case of doxorubicin, the IC50 values were found to be approximately five to six times more than those in the 2D cultures. The structural arrangement of spheroids can hinder treatment effectiveness, as diffusion gradients influence both oxygen availability and the distribution of chemotherapy agents. Unlike in 2D cultures, where doxorubicin and cisplatin are uniformly accessible to all cells, their penetration into the deeper layers of spheroids may be restricted, limiting their reach to centrally located cells [28,29]. This suggests that the 3D tumor architecture, characterized by enhanced extracellular matrix deposition and cellular interactions, contributes to drug resistance by limiting drug accessibility and altering cellular sensitivity [19,22,30].

While the development of collagen-embedded 3D spheroid models for breast and cervical cancers offers a promising platform for studying tumor biology and drug responses, certain limitations must be acknowledged. Although using collagen type I increases the likelihood of the model more closely mimicking in vivo conditions, it also restricts comparative analysis with other ECM compositions that may better reflect tissue-specific contexts. Additionally, the current model lacks stromal cell populations, such as fibroblasts or immune cells, which further limits its ability to fully represent the complexity of the TME. Furthermore, additional drug diffusion barriers due to collagen embedding, apart from the inherent 3D embedded spheroid architecture, may lead to elevated IC50 values, highlighting the challenges of chemotherapeutic penetration into solid tumors. Furthermore, additional histological studies to corroborate the findings of viability assays would undoubtedly enhance the results; however, this was beyond the scope of the current study.

Overall, this biomimetic model bridges the gap between traditional 2D cultures and in vivo conditions. This strategy improves translational research by enhancing the predictive capabilities of preclinical models, ultimately facilitating the development of personalized treatment approaches that consider tumor-specific features and microenvironmental factors. Further, this study also identifies areas for future refinement to improve the physiological accuracy of the development of spheroid models and the predictive capability of these models for drug screening platforms. Also, such findings highlight the necessity for advanced therapeutic strategies to overcome the limitations, including developing drug delivery systems that enhance drug penetration and efficacy within solid tumors.

## Conclusions

The collagen-embedded 3D spheroid model represents a robust platform for generating 3D cancer spheroids across various cell lines. The characterization, encompassing viability, growth dynamics, necrotic core formation, and overexpression of EMT markers, suggests an enhanced ability to replicate key features of in vivo solid tumors. Notably, the observed increase in IC50 values for anti-cancer drugs in 3D spheroids compared to traditional 2D cultures underscores the impact of 3D architecture on drug responsiveness. Given these attributes, this model holds significant promise not only for studying tumor biology but also for future applications in patient-derived tumor spheroid generation, advancing personalized cancer research, and therapeutic development.
